# Profile of Differential Promoter Activity by Nucleotide Substitution at GWAS Signals For Multiple Sclerosis

**DOI:** 10.1097/MD.0000000000000281

**Published:** 2014-12-02

**Authors:** Jihye Ryu, Jeyoung Woo, Jimin Shin, Hyunju Ryoo, Younyoung Kim, Chaeyoung Lee

**Affiliations:** From the School of Systems Biomedical Science, Soongsil University, 511 Sangdo-dong, Dongjak-gu, Seoul 156-743, Korea.

## Abstract

This experimental study was conducted with completely randomized design.

Genome-wide association studies (GWAS) have revealed a large number of genetic associations of nucleotide sequence variants with susceptibility to multiple sclerosis (MS). Nevertheless, studies to identify the functional relevance of these variants lag far behind identification of the GWAS signals. Expression quantitative trait loci (eQTLs) analysis and promoter activity analysis with the variants filtered by GWAS were conducted to identify their functional alleles and haplotypes. The promoter activity was assayed with reporter constructs containing variants at 8 MS GWAS signals resulted from 18 GWAS.

The promoter activity differed by alternative sequence variants at upstream regions of the *CYP24A1*, *CYP27B1*, *SYK*, *RAD21L1*, *PVR*, *ODF3B*, and *RGS14* genes (*P* < 0.05). The transcriptional regulations of sequence variants were also found by identifications of eQTLs for their corresponding genes with lymphoblastoid cells in the current study (*SYK*, *ODF3B*, *RGS14*, and *PVR*, *P* < 8.33 × 10^−3^) and with dendritic cells in a previous study (*CYP27B1*, *P* = 1.84 × 10^−6^).

This study identified regulatory nucleotide sequences in the promoters of the *CYP24A1*, *CYP27B1*, *SYK*, *RAD21L1*, *PVR*, *ODF3B*, and *RGS14* genes, and their variants differentially affected gene expression. This might result in their associations with MS susceptibility in previous GWAS. Further functional studies are required to understand the process of transcriptional regulation of the variants identified in the current study and the mechanisms underlying susceptibility to MS.

## Abstract 2

Supplemental Digital Content is available in the text

## INTRODUCTION

Multiple sclerosis (MS) is a complex inflammatory disease of the central nervous system that is accompanied by neurological impairment and disability. A number of linkage and association analyses found that the major histocompatibility complex (MHC) encoding the human leucocyte antigen genes on chromosome 6 confers a genetic influence on susceptibility to MS.^1^ However, involvement of any other loci had not been reproducibly demonstrated^2,3^ until the recent advance of genome-wide association studies (GWAS). GWAS not only identified associations of MS with common sequence variants outside the MHC region, but also confirmed the genetic contribution of the genes within the MHC region.^4^ Only a small proportion of the variants identified by GWAS were located in coding regions, and the functional relevance of most GWAS signals remains unknown. Thus, the functions of the variants in the GWAS signals should be determined to understand the mechanisms underlying development of MS. In this study, we investigated the differences in transcription activity resulting from nucleotide substitution in promoter sequences identified by GWAS.

## MATERIALS AND METHODS

### Selection of Promoter Variants

Candidate promoter nucleotide variants for association with MS were selected from the GWAS catalog (http://www.genome.gov/page.cfm?pageid=26525384&clearquery=1#result_table) and confirmed in the publications in which they were originally described. Genetic association signals were obtained from 18 GWAS for susceptibility to MS. We filtered the variants located within a region −2–0 Kb upstream of every gene encoding protein in order to examine the promoter activity. Linkage disequilibrium (LD) estimates were obtained with the variants within 2 Kb upstream of the transcription start site. We used genotypes of 379 Europeans at the variants provided by the 1000 Genomes Project (http://browser.1000genomes.org/). LD blocks were constructed for the variants associated with MS and variants linked to them using the algorithm of Gabriel et al^5^ (Haploview version 4.2) in which the block was defined according to the 95% confidence interval of the D′ value for pairwise LD between the variants with minor allele frequency > 0.05.

### Expression Quantitative Trait Loci (eQTLs) Analysis

We examined transcriptional regulation of the selected variants at GWAS signals by eQTL analysis. This analysis helped identify variants as *cis*-regulatory loci at a cell level by their transcriptional associations with the corresponding genes. Quantitative expression data from GEUVADIS RNA sequencing project were used (ftp://ftp.ebi.ac.uk/pub/databases/microarray/data/experiment/GEUV/E-GEUV-1/analysis_results/). They calculated expression level of each corresponding gene as the sum of all transcript reads per kilobase per million (RPKMs) using lymphoblastoid cell lines of 373 European individuals from the 1000 Genomes Project.^6^ The corresponding genotypes at the selected variants of the GWAS signals were obtained based on 1000 Genome Phase 1 (ftp://ftp.ebi.ac.uk/pub/databases/microarray/data/experiment/GEUV/E-GEUV-1/genotypes/). We identified eQTL using the simple linear regression model implemented in PLINK (v1.07). In order to reduce spurious associations produced by multiple testing, the Bonferroni correction was applied with the number of tested genes because of the linkage of the tested variants within each LD block.

### Plasmid Construction

**FIGURE 1:**
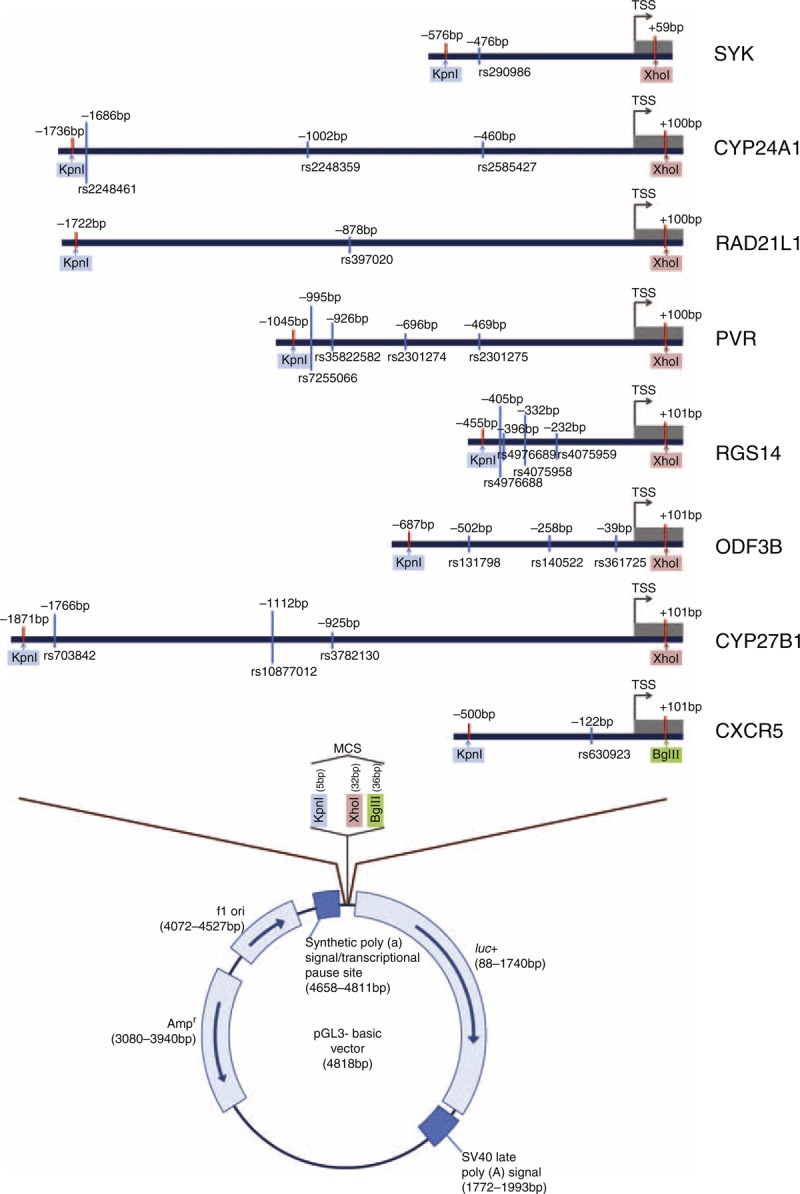
Schematic diagram of the luciferase reporter constructs. Horizontal bars are in the same scale. The sequences within the 2 red vertical bars of each reporter construct were inserted in the pGL3-Basic Vector. Light blue vertical bar indicate the sequence variants. Color box indicates restriction enzyme site. MCS, multiple cloning sites; TSS, transcription start site.

Luciferase reporter was constructed with the promoter variants as shown in Figure 1. The promoter region of each gene was amplified by polymerase chain reaction (PCR) with the primers designed in Supplementary http://links.lww.com/MD/A104. Restriction enzyme sites (Kpn I, Xho I, and BglII) were also added to each set of primers to facilitate posterior cloning of the PCR products (Figure 1). The PCR product covered the selected variants including downstream region (1–101 bp) of each gene. The PCR products were ligated into T-blunt vector (Promega, Madison, WI) using T4 DNA Ligase (Promega). The ligated plasmids were amplified using *E coli* DH5α. They were double-digested by Kpn I, Xho I, and BglII (NEB, Ipswich, MA) to release the fragments containing the promoter of each gene. The released fragments were subcloned upstream of the firefly luciferase reporter gene in the pGL3-Basic vector (Promega) using T4 DNA Ligase (Promega). Site-directed mutagenesis was performed to substitute appropriate nucleotide variants (QuickChange^™^ Site-Directed Mutagenesis Kit, Stratagene, La Jolla, CA). The sequences of all inserts were verified by direct sequencing.

### Transient Transfection

We selected the human embryonic kidney 293 (HEK293) cell line for luciferase reporter assay because the genes examined in the current study are all expressed in the human kidney. The HEK293 cell has been widely used in transient transfection for promoter activity experiments using plasmid vector because of its high transfection efficiency. HEK293 cells were cultured in Dulbecco's modified Eagle medium (Invitrogen, Carlsbad, CA) supplemented with 10% (v/v) heat-inactivated fetal bovine serum (Wellgene, Daegu, Korea), 100 units/mL Penicillin, and 100 μg/mL Streptomycin at 37°C in an atmosphere containing 5% CO_2_. Cells (1.6 × 10^5^) were seeded 24 hour prior to transfection in 6-well plates and transfected at approximately 60% to 70% confluence. Cells in each well were cotransfected with 1.2 μg pGL3-basic firefly luciferase reporter vector (Promega) containing the reporter construct and 24 ng pRL-CMV *Renilla* luciferase reporter vector (Promega) using Lipofectamine 2000 (Invitrogen) according to the manufacturer's instructions. Transfected cells were incubated for 24 hour at 37°C and 5% CO_2_. The amount of firefly and *Renilla* luciferases was measured sequentially from a single aliquot of cell lysate using the Dual-Glo Luciferase Assay system (Promega) on a CentroPRO LB962 luminometer (Berthold Technologies, Bad Wildbad, Germany). Transfection efficiency was controlled by screening experiments with the amount of *Renilla* luciferase within the range of ±30%. Firefly luciferase activity of each individual transfection was normalized to *Renilla* luciferase activity to correct for the difference in transfection efficiency. Promoter activity was expressed relative to that of the pGL3-Basic vector without the promoter sequence. All assays were conducted in triplicate, and at least 3 independent experiments were performed for each reporter gene construct.

### Prediction of Transcription Factor Binding Sites

Putative transcription factor binding sites (TFBS) at the promoter loci were predicted using PROMO version 3.0.2.^7^ Similarity of the query locus was scored by comparing with the positional weight matrix for each transcription factor, and the matrix was estimated based on real data by TRANSFAC^®^ version 8.3. We employed a similarity threshold value of 0.85. Changes in the predicted TFBS by allelic substitution were examined.

### Statistical Analysis

Statistical difference in luciferase activity among haplotypes was determined by one-way analysis of variance. Post hoc pairwise comparisons were performed with the false positive threshold of 0.05. We employed Tukey's honest significant difference test to compare all possible pairs of luciferase activity. All the statistical tests were conducted using the Statistical Package for the Social Sciences version 20 (SPSS Inc., Chicago, IL).

## RESULTS

We obtained 192 GWAS signals resulting from 18 GWAS for susceptibility to MS. The signals were located in 101 intragenic (Supplementary http://links.lww.com/MD/A104) and 91 intergenic (Supplementary http://links.lww.com/MD/A104) regions. Most variants were in regulatory regions, with 5 variants located in translated regions. Nine signals were located within the region −2–0 Kb upstream of genes. One signal was located in upstream regions of the genes encoding a microRNA (hsa-mir-548ac). Eight signals were promoter sequence variants of the genes encoding CYP24A1 (MIM 126065), CYP27B1 (MIM 609506), SYK (MIM 600085), RAD21L1, CXCR5 (MIM 601613), PVR (MIM 173850), ODF3B, and RGS14 (MIM 602513) proteins and were selected to examine their promoter activity. The GWAS signals were not in strong LD with any variants from transcription start site to +1 Kb of each gene. The odds ratio estimates for their associations with MS are summarized in Table 1.^8–10^

**TABLE 1:**
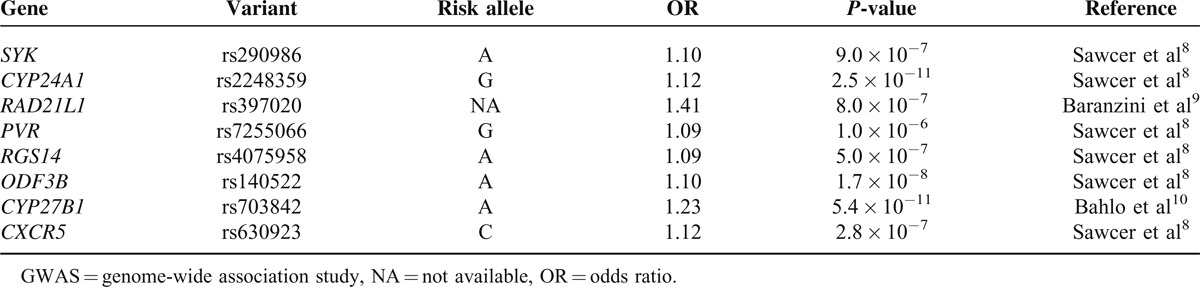
Candidate Promoter Nucleotide Variants Associated With Multiple Sclerosis Susceptibility in GWAS

**FIGURE 2:**
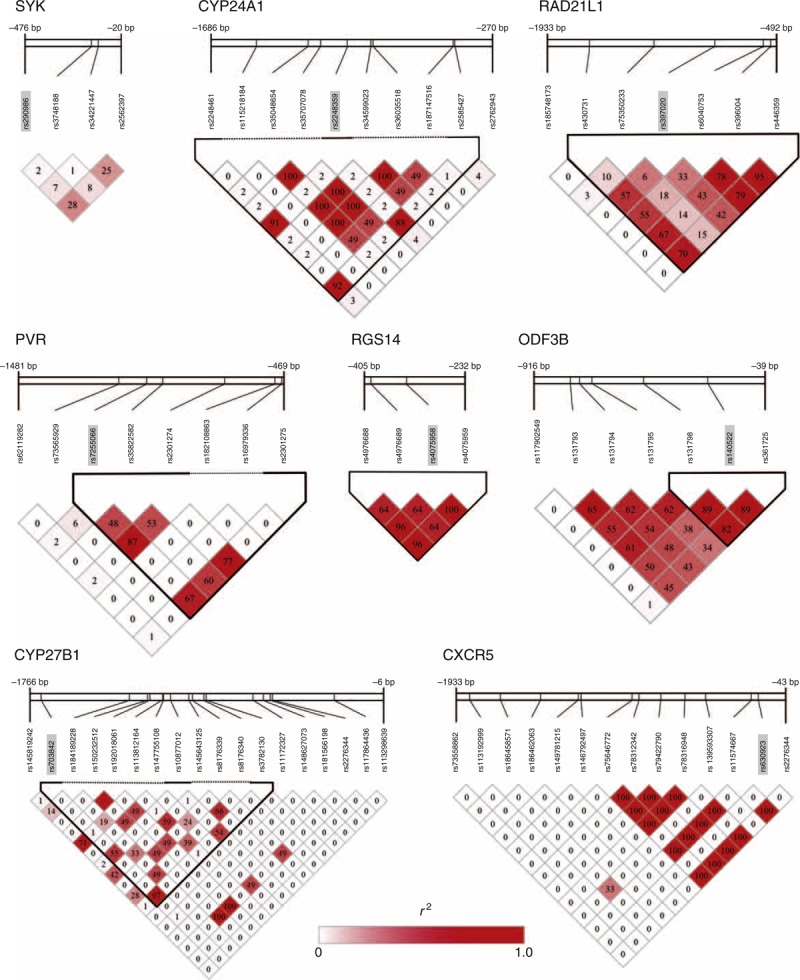
Linkage disequilibrium (LD) plots for promoter variants in the candidate regions of GWAS signals. The cells with color gradation present pairwise LD estimates (*r*^2^) and include their value among single nucleotide polymorphisms. The sequence variant associated with multiple sclerosis is presented on a shaded background, and the sequence variants in the same LD block are in bold. Reporter constructs included these variants to examine their transcriptional activity. The scale at the top of each image shows relative position of each sequence variant in promoter region. The negative values above the scale indicate the distance upstream of the transcription start site. Only the blocks examined in promoter assay are presented.

LD blocks (Figure 2) were constructed from linkage analyses using all variants located within the region −2-0 Kb upstream of each gene. Haplotype frequencies were estimated with the variants within LD blocks including GWAS signals (Table 2). We used only a single variant of some signals in the forward assay. The GWAS signals for *SYK* and *CXCR5* were not linked to any other promoter variants. Allelic substitution was successful at rs397020 in the upstream region of the *RAD21L1* gene; however, we failed to obtain proper polymorphic haplotypes because of highly repeated sequences in the region.

**TABLE 2:**
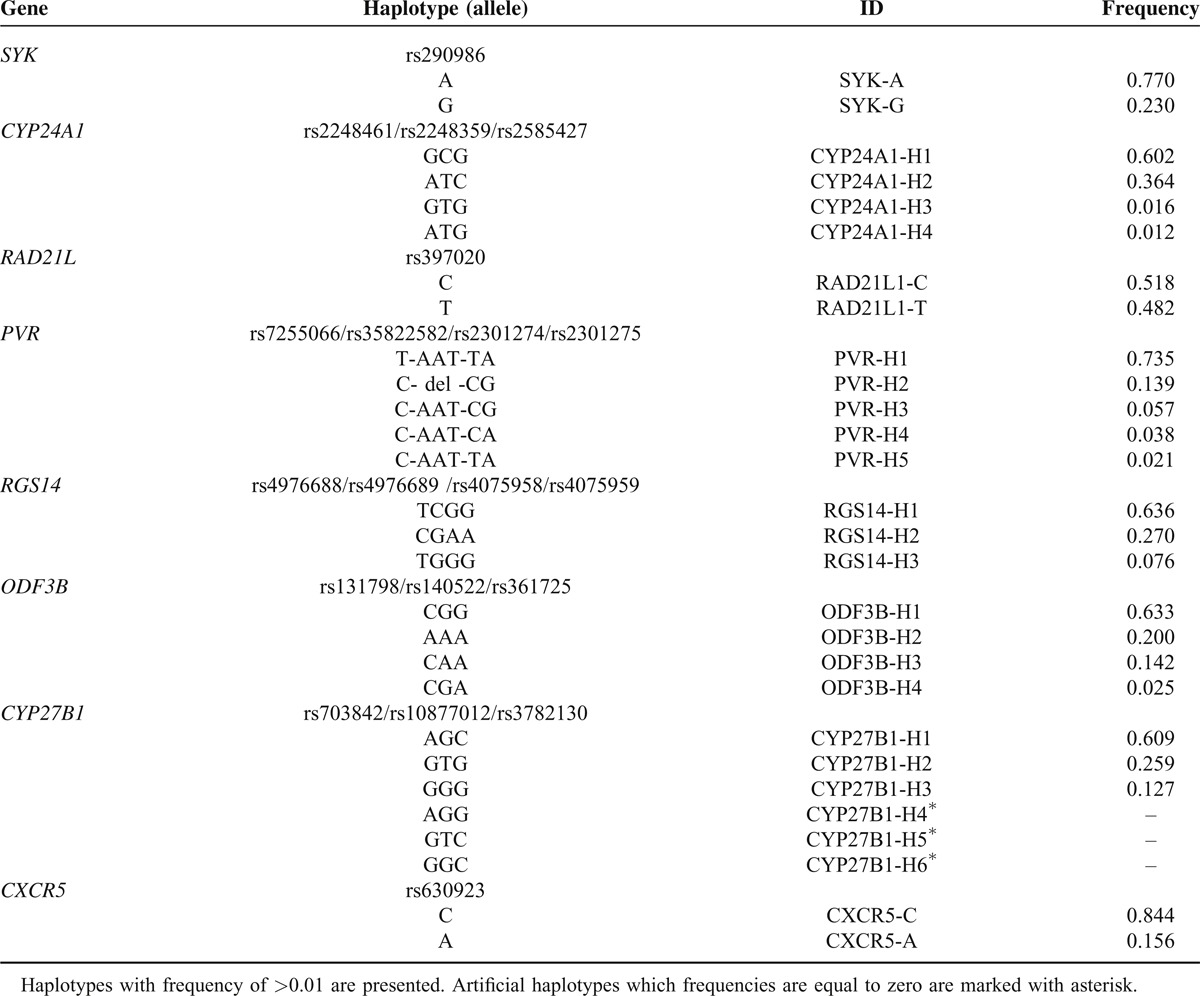
Haplotypes Containing Variants Associated With Multiple Sclerosis in Europeans

The eQTLs were identified by testing association with the expression level of 6 genes (*CYP27B1*, *CXCR5*, *SYK*, *ODF3B*, *RGS14*, and *PVR*) because the expression data of *CYP24A1* and *RAD21L1* were unavailable. The expression levels of *SYK*, *ODF3B*, and *RGS14* were significantly affected by the variants of the GWAS signals (Table 3, *P* < 8.33 × 10^−3^). Marginal associations were observed between *PVR* expression and its promoter variants (*P* < 0.05). The risk alleles resulted from GWAS studies increased the expression of *SYK*, *RGS14*, and *PVR* but decreased the expression of *ODF2B*.

**TABLE 3:**
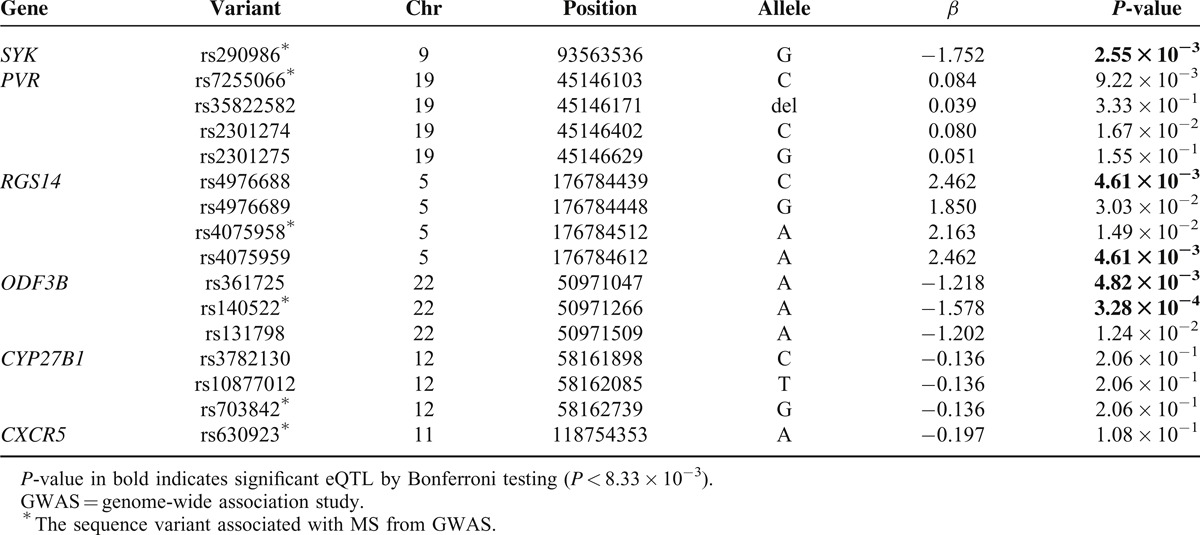
Associations of Promoter Variants at GWAS Signals With Expression of Their Corresponding Genes

**FIGURE 3:**
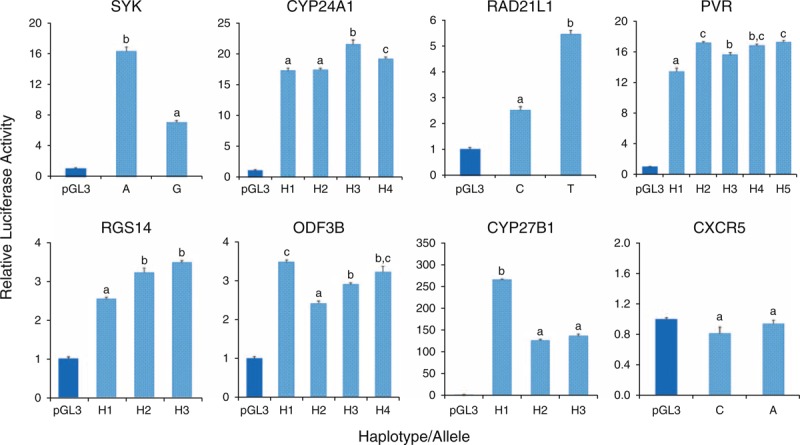
Promoter activity of reporter constructs containing candidate sequence variants associated with multiple sclerosis. The promoter activity of the pGL3 vector without sequence variants is shown in dark blue, and the activity of the vector containing promoter sequence variants is shown in light blue. Bars with the same lowercase letter indicate no significant difference among their promoter activities within each gene by the Tukey multiple comparison test (*P* > 0.05).

Differences in luciferase activity were found with reporter constructs for all genes, with the exception of *CXCR5* (Figure 3, *P* < 0.05). In particular, the expression of CYP27B1-H1, SYK-A, and RAD21L1-T was greater than 2-fold higher than that of CYP27B1-H2, SYK-G, and RAD21L1-C, respectively.

**FIGURE 4:**
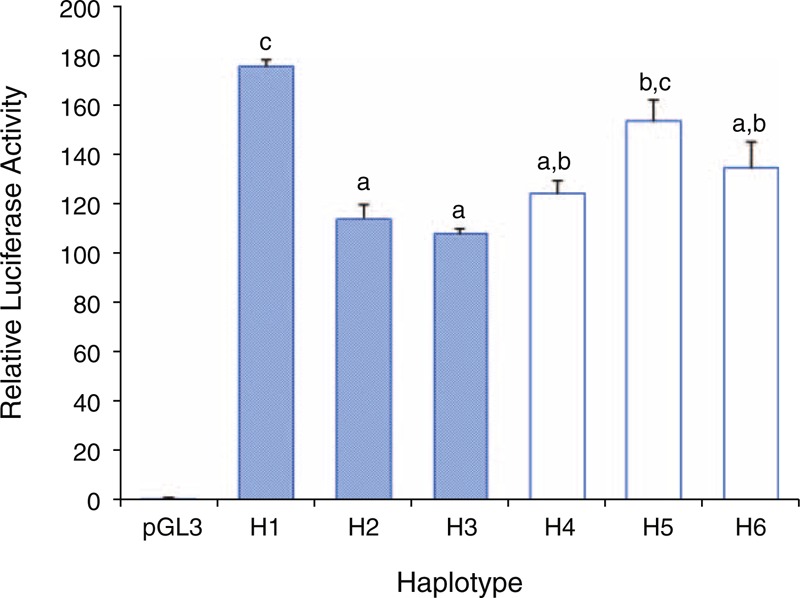
Transcriptional activity with artificial haplotypes of *CYP27B1*. Transcriptional activity of the pGL3 vector without sequence variants is shown in dark blue. Transcriptional activity with natural European haplotype is shown in light blue. Transcriptional activity with artificial haplotypes is shown in white. Bars with the same lowercase letter indicate no significant difference among their promoter activities by the Tukey multiple comparison test (*P* > 0.05).

Luciferase activity differed significantly with only a single nucleotide substitution for reporter constructs of all 7 genes. Differential luciferase activity of 1 alternative allele was observed with natural haplotype constructs for each of 6 genes (Figure 3). Luciferase analysis with additional artificial haplotypes of *CYP27B1* revealed that the alteration in expression was the result of a nucleotide substitution at 1 SNP (rs3782130) within the LD block (Figure 4, *P* < 0.05).

In silico prediction showed allele-specific TFBS at the nucleotide variants examined in the current study (Supplementary http://links.lww.com/MD/A104). In particular, the variants of *CYP27B1*, *SYK*, and *RAD21L1* that were found to be largely different in promoter activity were also predicted to have largely different TFBS by allele substitution. No different TFBS were predicted by nucleotide substitution of only rs140522 (Supplementary http://links.lww.com/MD/A104).

## DISCUSSION

We found that GWAS signals identified for susceptibility to MS by recent GWAS were located primarily in noncoding genomic regions. Although these signals may include candidate functional variants for regulation of genes predisposing to MS, there have been no trials investigating their functions. The current study first identified functional variants for susceptibility to MS, focusing on aberrant function of all promoter regions filtered by GWAS.

This study was successful in identifying functional sequence variants in 7 promoter regions. Six SNPs associated with MS were causal variants (*CYP24A1*, *CYP27B1*, *SYK*, *RAD21L1*, *PVR*, and *ODF3B*), and 4 of the 6 SNPs shared functions with their linked SNPs (*CYP24A1*, *CYP27B1*, *PVR*, and *ODF3B*). One SNP was linked to a causal SNP (*RGS14*). The transcriptional regulations of the variants were supported by the eQTL analysis. The promoter variants were identified as eQTLs for the expression of the genes encoding SYK, ODF3B, RGS14, and PVR in lymphoblastoid cells. The directions of allelic effects from the promoter assay were concurred with those from the eQTL analysis. The promoter variant of *CYP27B1* was reported as an eQTL from the previous study with human dendritic cells (rs703842, *P* = 1.84 × 10^−6^).^11^

Promoter activity was not differentiated by nucleotide substitutions of the *CXCR5* promoter. Since the promoter activity was small in the HEK293 cells, the inference on the negligible difference cannot be extended to other cell types. The eQTL analysis with lymphoblastoid cells showed that any eQTL was not identified for *CXCR5*. This association resulted from GWAS might be only a false positive or be caused by a function other than promoter activity. A lesson learned from the current functional study is that the genetic associations identified by GWAS are quite meaningful. The concordant observations from the promoter activity analysis and eQTL analysis suggested that the nucleotide substitutions at the GWAS signals are likely to affect expression levels of the corresponding genes in human immune cells. Therefore, we would encourage others to conduct intensive GWAS to identify sequence variants for susceptibility to MS.

Surprisingly, transcriptional activity was altered by a single nucleotide substitution. In particular, the G allele of the *SYK* variant and the T allele of the *RAD21L1* variant drove greater than 2-fold higher transcriptional activity than SYK-A and RAD21L1-C, respectively. Of course, we also found an influence of multiple nucleotide variants on transcriptional activity. For example, the largest difference in transcriptional activity was found between the major haplotype and the haplotype with complementary nucleotides, with the transcriptional activity of the other haplotypes falling between these 2 values (see *CYP27B1*, *ODF3B*, and *PVR*).

The identified variants may influence transcription of genes that play important roles in the immune system and, thus, in MS. CYP27B1 produces active vitamin D (1,25-hydroxyvitamin D (1,25 (OH)_2_ D)) in immune and central nervous system cells, and CYP24A1 catalyzes the conversion of active vitamin D to the inactive form (24,25 (OH)_2_ D).^12,13^ Because vitamin D is an important environmental factor in treating MS, CYP27B1 and CYP24A1 have been known to be factors in susceptibility to MS, based on control of vitamin D level.^14^ SYK is a tyrosine kinase involved in signal transduction in a variety of immune cells,^15^ and aberrant regulation of SYK is associated with autoimmune diseases, such as rheumatoid arthritis^16^ and lupus^17^. PVR regulates check points to activate T cells in the central nervous system.^18^*RGS14* and *ODF3B* were identified as TNF-κB p65 target genes in human B lymphoblastoid cells using ChIP-Seq.^19^

Inflammation is another potential function of the genes that may predispose to MS. For example, CYP27B1 and CYP24A1 perturb active vitamin D level and, thus, enhance production of proinflammatory cytokines and chemokines. SYK is essential for the transmission of inflammatory signals of various cytokines and influences pathophysiology of inflammatory diseases, such as atherosclerosis^20^ and vascular dementia.^21^

Specific mechanisms underlying the different promoter activities observed in this study are not known. Nevertheless, we suspect that nucleotide substitutions would change TFBS in a variety of ways. Our in silico prediction showed that most alternative alleles examined in this study potentially induce changes in TF binding. Further research is needed to identify genuine TF profiles by allelic substitutions. We found that a predicted site was a true TFBS using ChIP-Seq data provided by ENCODE consortium.^22^ The TFBS encompassing rs2585427 upstream of the *CYP24A1* gene was identified as binding to E2F-1 in HeLa-S3 cells. Difference resulting from allele substitution, however, remains unknown. Promoter activity of the variants examined in the current study was also supported by various evidences of regulatory functions provided by RegulomeDB.^23^ For example, most of the variants turned out to have open chromatin structure as shown in Supplementary http://links.lww.com/MD/A104, which indicates potential regulatory sequences.

We found differential transcriptional activity in promoter regions of the *CYP24A1*, *CYP27B1*, *SYK*, *RAD21L1*, *PVR*, *ODF3B*, and *RGS14* genes. This is the first study to identify functional nucleotide variants associated with many GWAS signals for MS on a large scale. The results suggest that alternative single nucleotides and/or haplotypes of the sequence variants may produce modified TFBS. We suspect that the promoter GWAS signals for MS might be the result of different transcriptional activity. Further functional studies are needed to identify the mechanisms underlying the association with development of MS. Functional studies are also required for MS GWAS signals in regions other than the promoter to simultaneously apply the genetic risk/protective factors for MS to personalized medicine.
